# Supplementary material for: Perceived community alignment increases information sharing
Source: Nat Commun. 2025 Jul 1;16:5864. doi: 10.1038/s41467-025-59915-8 (PMC12215788; doi:10.1038/s41467-025-59915-8)
Supplement: Supplementary file 1 — Supplementary Information [file 41467_2025_59915_MOESM1_ESM.docx]

Supplementary Information for **“Perceived community alignment increases information sharing”**

^1^Department of Psychology, University of Southern California, Los Angeles, CA, United States of America, ^2^Department of Psychology, University of California, Los Angeles, Los Angeles, CA, United States of America, ^3^Department of Mathematics, University of California, Los Angeles, Los Angeles, CA, United States of America, ^4^Department of Sociology, University of California, Los Angeles, Los Angeles, CA, United States of America, ^5^Sante Fe Institute, Santa Fe, NM, United States of America, ^6^Brain Research Institute, University of California, Los Angeles, Los Angeles, CA, United States of America

* Corresponding authors:

Elisa C. Baek: [elisa.baek@usc.edu](mailto:elisa.baek@usc.edu)

Carolyn Parkinson: [cparkinson@ucla.edu](mailto:cparkinson@ucla.edu)

**Supplementary Table 1: Descriptions of stimuli**

Supplementary Table 1. Descriptions of stimuli

|  | Video | Content |
| --- | --- | --- |
| 1 | An Astronaut’s View of Earth | An astronaut discusses viewing Earth from space and, in particular, witnessing the effects of climate change from space. He then urges viewers to mobilize to address this issue. |
| 2 | All I Want | A sentimental music video depicting a social outcast with a facial deformity who is seeking companionship. |
| 3 | Scientific demonstration | An astronaut at the International Space Station demonstrates and explains what happens when one wrings out a waterlogged washcloth in space. |
| 4 | Food Inc. | An excerpt from a documentary discussing how the fast-food industry influences food production and farming practices in the United States. |
| 5 | We Can Be Heroes | An excerpt from a mockumentary-style series in which a man discusses why he nominated himself for the title of Australian of the Year. |
| 6 | Ban College Football | Journalists and athletes debate whether football should be banned as a college sport. |
| 7 | Soccer match | Highlights from a soccer match. |
| 8 | Ew! | A comedy skit in which grown men play teenage girls disgusted by the things around them. |
| 9 | Life’s Too Short | An example of a ‘cringe comedy’ in which a dramatic actor is depicted unsuccessfully trying his hand at improvisational comedy. |
| 10 | America’s Funniest Home Videos | A series of homemade video clips that depict examples of unintentional physical comedy arising from accidents. |
| 11 | Zima Blue | A philosophical, animated short set in a futuristic world. |
| 12 | Nathan For You | An episode from a ‘docu-reality’ comedy in which the host convinces people, who are not always in on the joke, to engage in a variety of strange behaviors. |
| 13 | College Party | An excerpt from a film depicting a party scene in which a bashful college student is pressured to drink alcohol. |
| 14 | Eighth Grade | Two excerpts from a film that depict a young teenager who video blogs about her mental-health issues and an awkward scene between two teenagers on a dinner date. |

Note: These videos were used in prior studies^1,2^; the descriptions of them in the present paper are the same as those in the prior studies.

**Supplementary methods 1 for analyses in Study 1**

**Permutation test for sharing likelihood and video order.** As we noted in the main manuscript, all participants saw the videos in the same order. To address potential concerns that video order may affect sharing likelihood, we conducted a permutation test. Specifically, while holding the sharing likelihood constant, we uniformly randomly shuffled the order of the videos 10,000 times. For each permutation of the data set, we calculated the Spearman rank correlation between the sharing likelihood and the labels that correspond to video order. This calculation generated an estimate of a null distribution of 10,000 Spearman correlation values that corresponds to what one would obtain by chance. We then computed a *p*-value by calculating the frequency with which the observed Spearman correlation between video order with sharing likelihood exceeded the Spearman correlation value in the null distribution. The resulting Spearman correlation value of 0.054 does not differ from what one would expect based on chance, with a *p*-value of 1.000.

**Supplementary table for Study 1 results: Subcortical results**

Supplementary Table 2. Results that relate ISCs with the binarized sharing variable: Subcortical results

Contrast: ISC_{high sharing, high sharing}_ > ISC_{low sharing, low sharing}_

| Subcortical region | β | 95% CI | *p* |
| --- | --- | --- | --- |
| Nucleus Accumbens (L) | –0.028 | [–0.095, 0.039] | 1.000 |
| Amygdala (L) | 0.035 | [–0.065, 0.135] | 1.000 |
| Caudate Nucleus (L) | –0.061 | [–0.141, 0.019] | 1.000 |
| Hippocampus (L) | 0.005 | [–0.089, 0.099] | 1.000 |
| Pallidum (L) | –0.056 | [–0.115, 0.003] | 1.000 |
| Putamen (L) | –0.084 | [–0.153, 0.015] | 0.551 |
| Thalamus (L) | 0.021 | [–0.036, 0.078] | 1.000 |
| Nucleus Accumbens (R) | 0.006 | [–0.09, 0.102] | 1.000 |
| Amygdala (R) | –0.025 | [–0.109, 0.059] | 1.000 |
| Caudate Nucleus (R) | –0.026 | [–0.116, 0.064] | 1.000 |
| Hippocampus (R) | 0.034 | [–0.013, 0.081] | 1.000 |
| Pallidum (R) | –0.025 | [–0.072, 0.022] | 1.000 |
| Putamen (R) | –0.105 | [–0.183, –0.027] | 0.044 |
| Thalamus (R) | 0.049 | [–0.027, 0.125] | 1.000 |

We Holm–Bonferroni-corrected all *p*-values due to multiple comparisons. The quantity β is the standardized regression coefficient, and CI is the confidence interval. The statistical tests are two-tailed.

**Supplementary figure for Study 1 results: Results of exploratory contrasts**


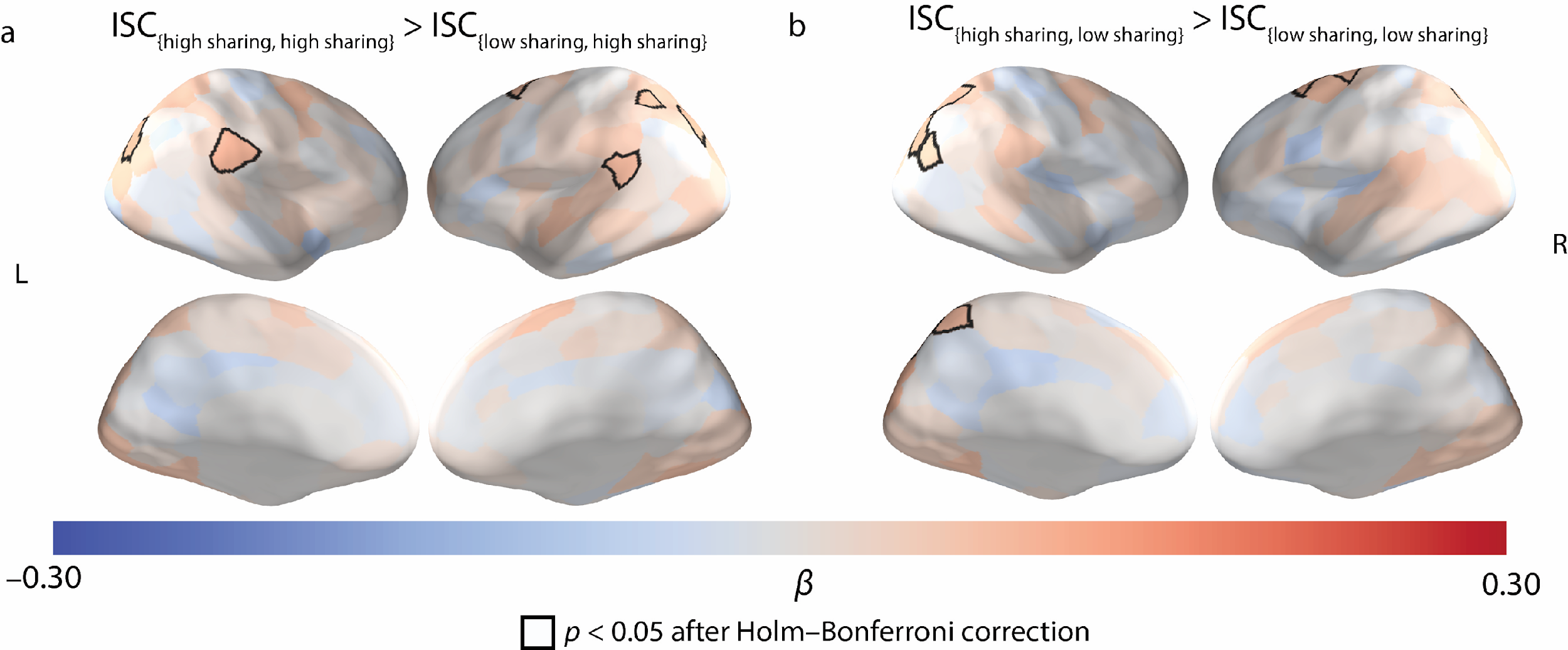


**Supplementary Fig 1. Additional exploratory contrasts that relate neural similarity with sharing likelihood. (a)** Similar to the results that we found for our primary contrast (i.e., ISC_{high sharing, high sharing}_ > ISC_{low sharing, low sharing}_; see Fig. 1c in the main manuscript), we observed larger ISCs in the temporoparietal junction, the superior parietal cortex, and portions of the visual cortex when both participants were very likely to share content than when one participant was very likely to share content and the other participant was unlikely to share content (i.e., ISC_{high sharing, high sharing}_ > ISC_{low sharing, high sharing}_). **(b)** We also obtained similar results for the ISC_{low sharing, high sharing}_ > ISC_{low sharing, low sharing}_ contrast, with larger ISCs in portions of the visual cortex and superior parietal cortex when one participant was very likely to share content than when both participants were unlikely to share content. The quantity β is the standardized regression coefficient. We used two-tailed statistical tests and employed Holm–Bonferroni correction to correct for multiple comparisons across brain regions. The authors created the brain visualizations using FreeSurfer, which is an open-source neuroimaging toolkit for processing, analyzing, and visualizing human brain magnetic-resonance images. https://surfer.nmr.mgh.harvard.edu/fswiki/FreeSurferSoftwareLicense.

**Supplementary figure for Study 1 results: Results using a non-binarized version of the sharing-likelihood ratings**

In our primary analyses (which we reported in the main manuscript) of data from Study 1, we binarized our sharing-likelihood variable. The original sharing-likelihood variable was on a 1–5 Likert scale (with “1 = very unlikely” and “5 = very likely”). In our binarization, we classified ratings of 3 or more as a “high sharing likelihood” and ratings of 2 or less as a “low sharing likelihood”. We also conducted analyses to test for associations between ISCs and a non-binarized version of the sharing-likelihood variable. To relate the participant-level sharing likelihood measure to the dyad-level neural-similarity measure, for each unique pair of participants, we first calculated a dyad-level variable that summarizes the overall likelihood of sharing each video by summing the sharing-likelihood ratings of both participants in a dyad. For example, if one member of a dyad rates their likelihood to share a video as “1” and the other member of the dyad rates their sharing likelihood as “4”, then the dyad-level variable for sharing has the value 5. We then took an analogous approach to the one that we described in the Methods and Results sections for Study 1 results in the main manuscript. Specifically, for each of our 214 brain regions, we fit a linear mixed-effects model with crossed random effects with the ISC in the corresponding region as the dependent variable, the dyad-level non-binarized sharing-likelihood variable as the independent variable, and similarities in participants’ age, gender, and country of origin as control variables. (See the Methods section of the main manuscript for more details on how we determined these control variables.) We also included random intercepts for each individual in a dyad, video, and interaction between each participant and each video. The models gave similar results (see Supplementary Fig. 2) as those that we reported in the main manuscript.


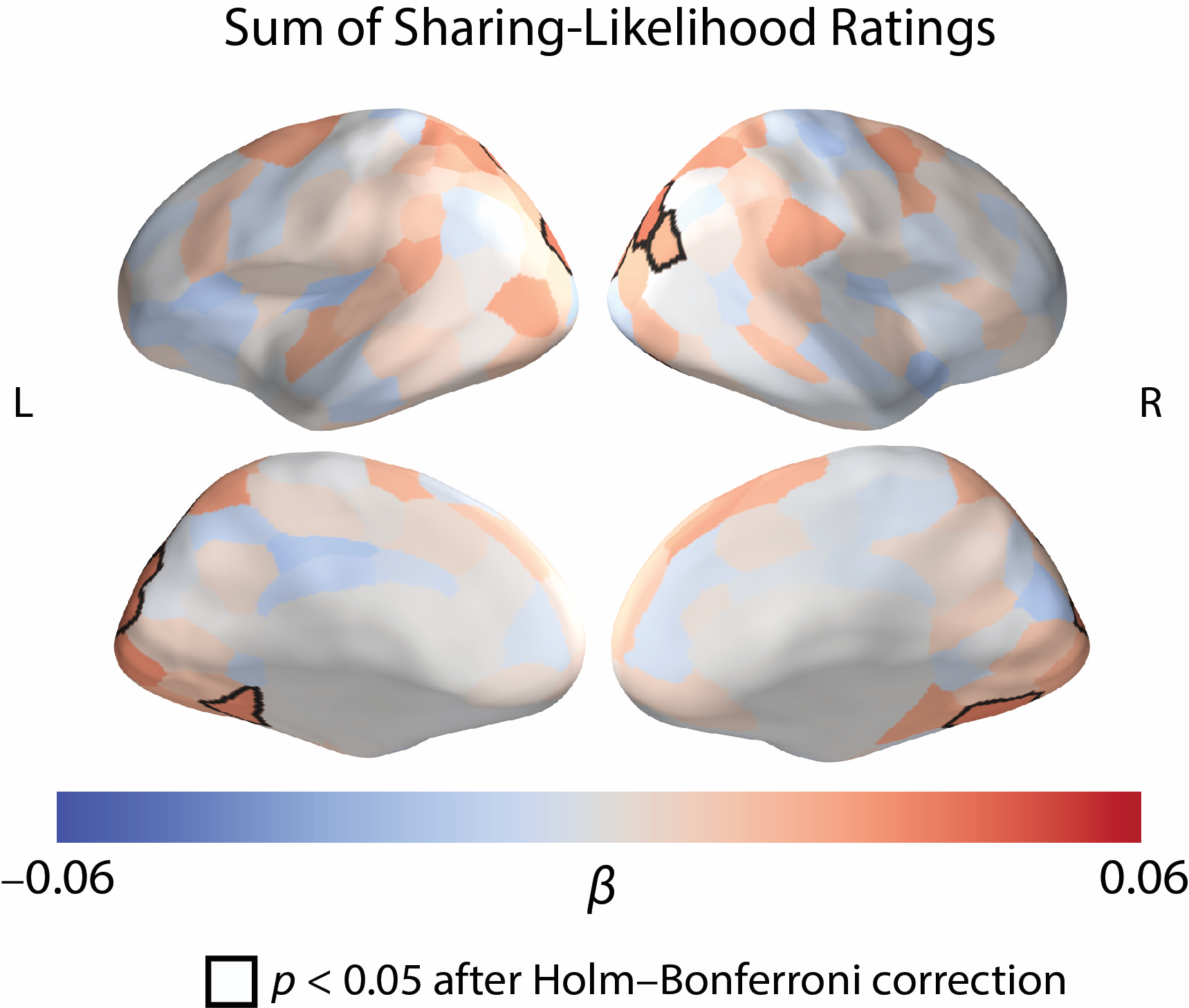


**Supplementary Fig 2. Relating neural similarity and sharing likelihood using a non-binarized variable to summarize sharing likelihood for members of each dyad. (a)** We obtained similar patterns in the results when using a non-binarized version of the sharing-likelihood variable (which equals the sum of the sharing-likelihood ratings of the two participants in a dyad) as in the results that we obtained when we related a binarized version of the sharing-likelihood variable with ISCs (i.e., ISC_{high sharing, high sharing}_ > ISC_{low sharing, low sharing}_; see Fig. 1c in the main manuscript). The quantity β is the standardized regression coefficient. We used two-tailed statistical tests and employed Holm–Bonferroni correction to correct for multiple comparisons across brain regions. The authors created the brain visualizations using FreeSurfer, which is an open-source neuroimaging toolkit for processing, analyzing, and visualizing human brain magnetic-resonance images. https://surfer.nmr.mgh.harvard.edu/fswiki/FreeSurferSoftwareLicense.

**Supplementary figure for Study 1 results: Alternative statistical-modeling approach**

To test the robustness of our results to alternative statistical-modeling approaches, we also conducted a modified version of the Mantel test of our primary analyses. First, we permuted the data 5000 times to create a null distribution of the data that accounts for the dependence structure of the data from repeating subjects and videos. Specifically, for each permutation, we first uniformly randomly shuffled the subject identifier while holding the brain data constant. We then uniformly randomly shuffled the video identifier while holding the brain data constant. We then ran the mixed-effects model that we reported in the main manuscript. For each permutation, we added the maximum *t*-statistic across regions and contrasts to a null distribution. We then compared the actual *t*-statistics from the un-permuted data to those from the null distribution. We show the results of this procedure in Supplementary Fig. 3. The results from this permutation-based approach are similar to our findings with the method that we reported in the main manuscript, although fewer parcels emerged as significant using this approach.

We also attempted to model our data using the Bayesian multilevel-modeling approach of Chen et al.^3^. However, due to the complexity of our data structure and the large number of observations, it was not computationally feasible to deploy this Bayesian approach with the available resources.

**
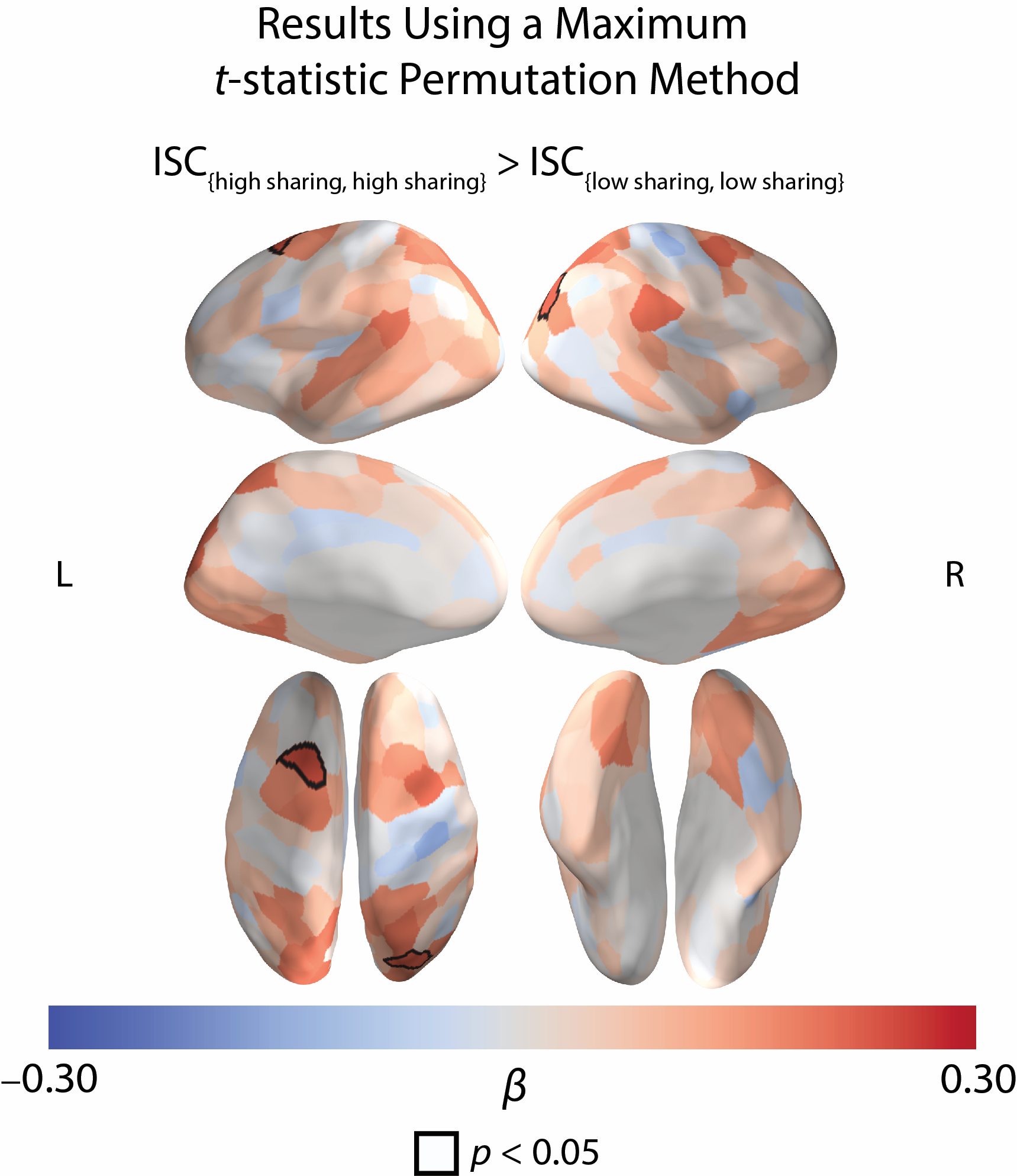
**

**Supplementary Fig 3. Relating neural similarity and sharing likelihood using a modified Mantel test with a maximum *t*-statistic approach.** Using this alternative statistical-modeling approach, we obtained similar patterns in the results to the ones that we reported in the main manuscript. The quantity β is the standardized regression coefficient. The statistical tests are two-tailed. The authors created the brain visualizations using FreeSurfer, which is an open-source neuroimaging toolkit for processing, analyzing, and visualizing human brain magnetic-resonance images. https://surfer.nmr.mgh.harvard.edu/fswiki/FreeSurferSoftwareLicense.

**Supplementary figure for Study 1 results: Results with matching numbers of observations**

Our dyad-level sharing-likelihood ratings had different numbers of observations for each of the three levels. Specifically, of the 29,770 unique pairs of ratings, 3485 were {high sharing, high sharing} pairs, 14,963 were {low sharing, low sharing} pairs, and 11,193 were {low sharing, high sharing} pairs. We tested the robustness of the effects that we reported in the main manuscript to account for the different numbers of observations. To do this, we first undersampled the data uniformly at random from the {low sharing, low sharing} and {low sharing, high sharing} observations to match the number of {high sharing, high sharing} observations. We then fit our main model on this portion of the data set with matching observations across the different levels of the sharing variable. We repeated this process 1000 times. We then averaged across the 1000 estimates for the contrast of interest (namely, {high sharing, high sharing} > {low sharing, low sharing}). We show the results in Supplementary Fig. 4. As the figure indicates, the results of these analyses closely resemble the results that we reported in the main manuscript.


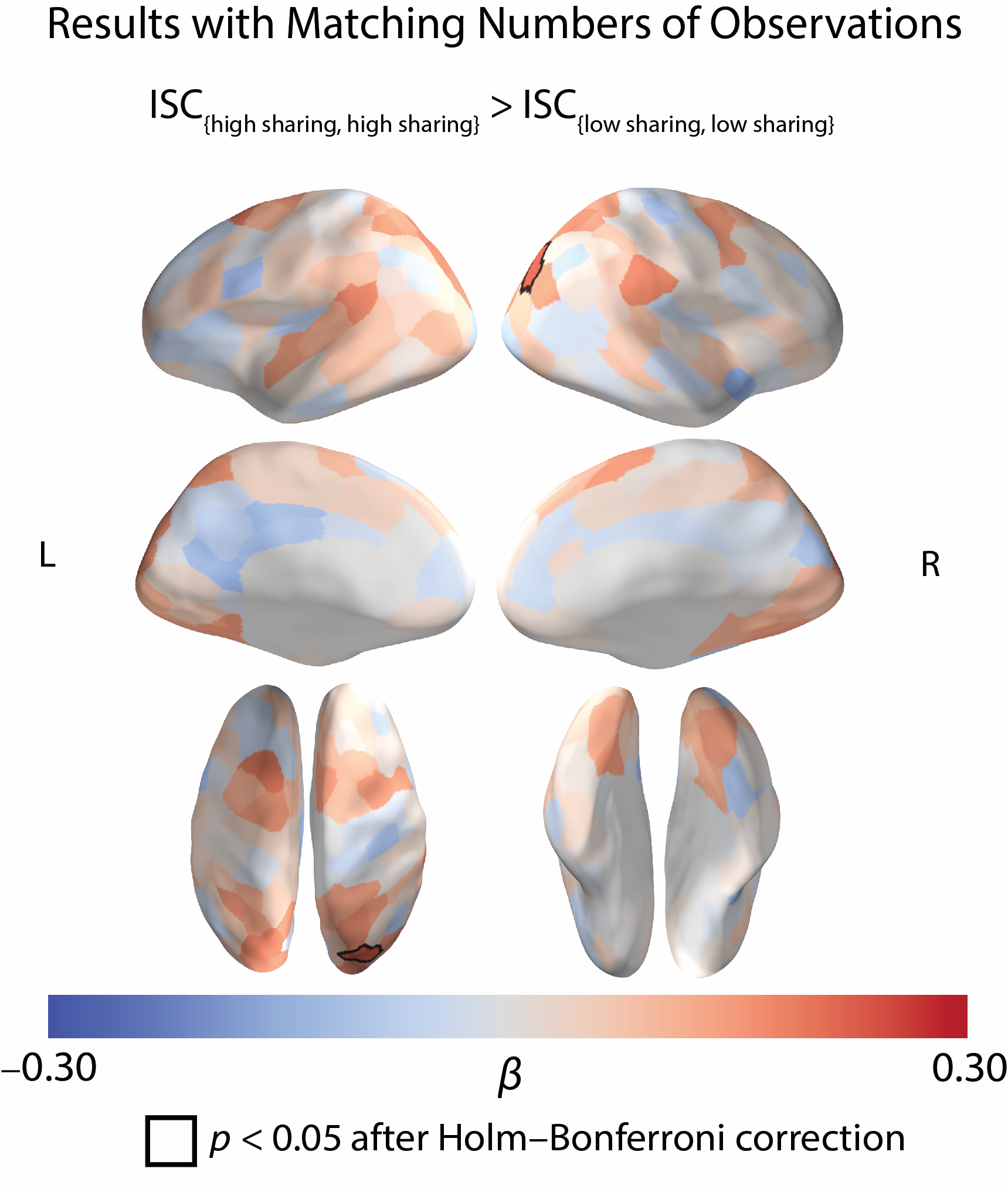


**Supplementary Fig 4. Relating neural similarity and sharing likelihood with matching numbers of observations.** The results that we obtained using subset data with matching observations of our dyad-level sharing-likelihood variable are similar to the results that we reported in the main manuscript. The quantity β is the standardized regression coefficient. We used two-tailed statistical tests and employed Holm–Bonferroni correction to correct for multiple comparisons across brain regions. The authors created the brain visualizations using FreeSurfer, which is an open-source neuroimaging toolkit for processing, analyzing, and visualizing human brain magnetic-resonance images. https://surfer.nmr.mgh.harvard.edu/fswiki/FreeSurferSoftwareLicense.

**Supplementary figure for Study 2 results: Results from permutation tests**

To test the robustness of the results of Study 2 to alternative statistical-modeling approaches, we also conducted a modified version of the Mantel test of our analyses. First, we permuted the data 1000 times to create a null distribution of the data that accounts for the dependence structure of the data from repeating subjects and articles. Specifically, for each permutation, we first uniformly randomly shuffled the subject identifier while holding the sharing data constant. We then uniformly randomly shuffled the video identifier while holding the sharing data constant. We then fit the mixed-effects model that we reported in the main manuscript. For each permutation, we added our estimate of the standardized regression coefficient β to a null distribution. We then compared the regression-coefficient estimate from the unpermuted data to those from the null distribution. We show the results of this procedure in Supplementary Fig. 5a. The results from this permutation-based approach confirm that the true model’s β value is significantly larger than the permuted β values, with a *p*-value of *p* < 0.001. We also performed an analogous modified Mantel test of our analysis when controlling for interest and valence ratings (see Supplementary Fig. 5b).


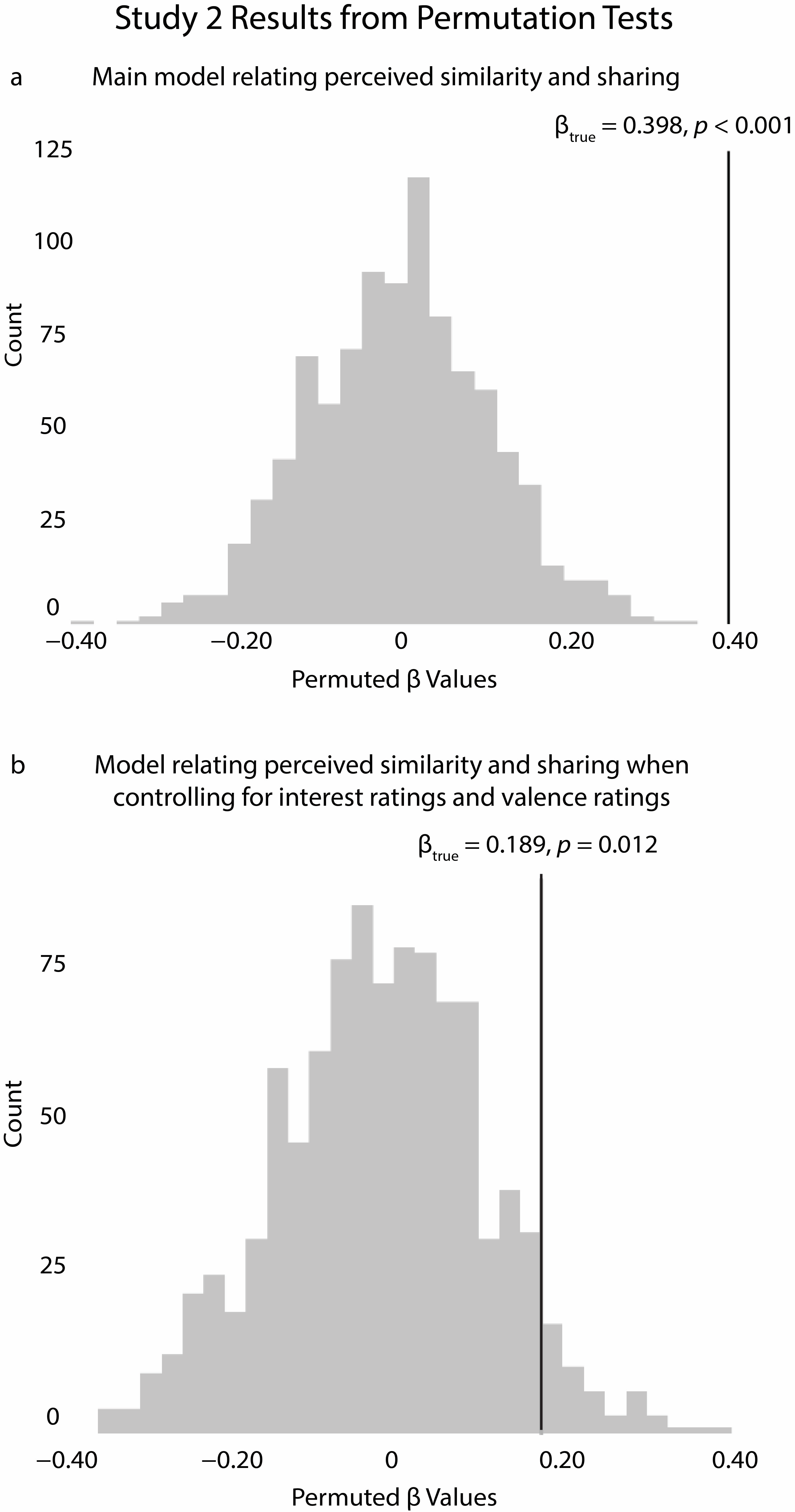


**Supplementary Fig 5. Permutation-test results of Study 2.** **(a)** The results that we obtained using a permutation test confirm that the value of the standardized regression coefficient β in the true model is significantly larger than the permuted β values, with a *p*-value of *p* < 0.001. **(b)** When controlling for interest ratings and valence ratings, the results that we obtained using a permutation test suggest that the true model’s β value is significantly larger than the permuted β values, with a *p*-value of *p* = 0.012. Each permutation test uses 1000 permutations of the data. The gray bars indicate the distribution of the permuted β values, and the black line indicates the true β value.

**Supplementary tables for Study 3 results: Results of all contrasts**

Supplementary Table 3. Results of Study 3 for all contrasts for predicting sharing likelihood

| Contrast | β | 95% CI | *p* |
| --- | --- | --- | --- |
| Similar > Dissimilar | 0.572 | [0.262, 0.882] | 0.001 |
| Similar > Mixed | 0.289 | [–0.021, 0.599] | 0.092 |
| Similar > Unclear | 0.583 | [0.271, 0.895] | 0.001 |
| Mixed > Dissimilar | 0.283 | [–0.029, 0.595] | 0.092 |
| Unclear > Dissimilar | –0.012 | [–0.326, 0.302] | 0.943 |
| Mixed > Unclear | 0.294 | [–0.02, 0.608] | 0.092 |

We FDR-corrected all *p*-values due to multiple comparisons. The quantity β is the standardized regression coefficient, and CI is the confidence interval. The statistical tests are two-tailed.

Supplementary Table 4. Results of Study 3 for all contrasts for predicting sharing likelihood when

controlling for interest ratings and baseline sharing ratings

| Contrast | β | 95% CI | *p* |
| --- | --- | --- | --- |
| Similar > Dissimilar | 0.854 | [0.478, 1.23] | < 0.001 |
| Similar > Mixed | 0.534 | [0.158, 0.910] | 0.012 |
| Similar > Unclear | 0.754 | [0.376, 1.132] | < 0.001 |
| Mixed > Dissimilar | 0.320 | [–0.058, 0.698] | 0.147 |
| Unclear > Dissimilar | 0.010 | [–0.372, 0.392] | 0.608 |
| Mixed > Unclear | 0.220 | [–0.162, 0.602] | 0.312 |

We FDR-corrected all *p*-values due to multiple comparisons. The quantity β is the standardized regression coefficient, and CI is the confidence interval. The statistical tests are two-tailed.

**Supplementary figure for Study 3 results: Results from a permutation test**

To test the robustness of our results to alternative statistical-modeling approaches, we also conducted a modified version of the Mantel test of our analyses of the Study 3 results. First, we permuted the data 1000 times to create a null distribution of the data. Specifically, for each permutation, we first uniformly randomly shuffled the sharing data while holding the condition labels constant. We then fit the linear-regression model that we reported in the main manuscript. For each permutation, we added our estimate of the standardized regression coefficient β to a null distribution. We then compared the regression-coefficient estimate from the unpermuted data to those from the null distribution. We show the results of this procedure in Supplementary Fig. 6. The results of this permutation test confirm that the true model’s β value is significantly larger than the permuted β values, with a *p*-value of *p* = 0.005.

**
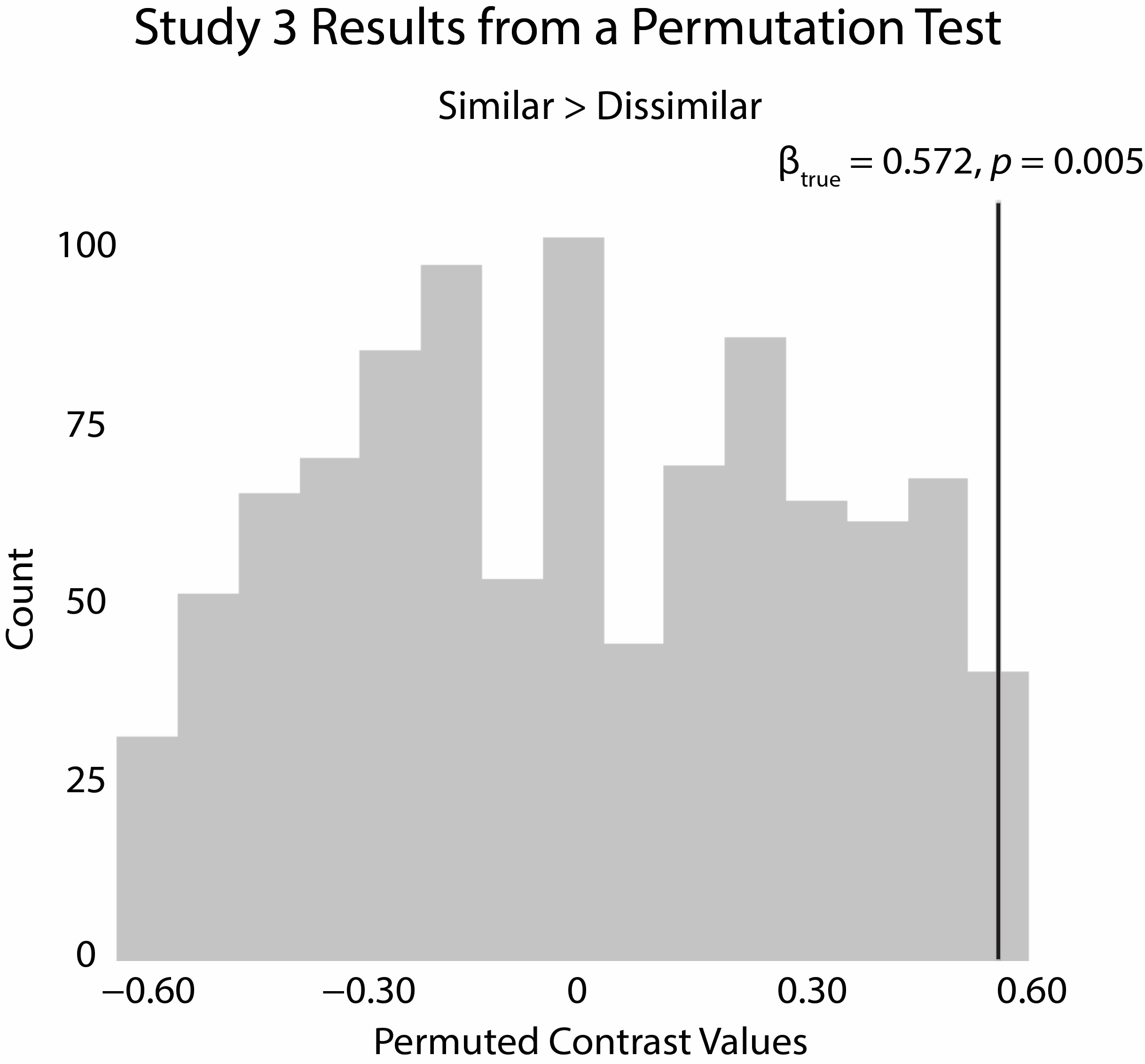
**

**Supplementary Fig 6. Permutation-test results of Study 3.** The results that we obtained with a permutation test confirm that the value of the standardized regression coefficient β in the true model is significantly larger than the permuted β values, with a *p*-value of *p* = 0.005. The permutation test uses 1000 permutations of the data. The gray bars indicate the distribution of the permuted β values, and the black line indicates the true β value.

**Supplementary table for Study 3 methods: Instructions for each experimental condition**

Supplementary Table 5. Instructions for the different participant groups in Study 3

| Condition | Instructions |
| --- | --- |
| Similar | Now, imagine that you are invited to a group on Facebook by your colleagues.    When you join, you realize that the majority of people in this group are **similar to you** in your likes and dislikes about the things that you just provided your answers to. In other words, they share your sense of humor, favorite types of movies to watch, how they spend their free time, as well as in ideology and political leanings. |
| Dissimilar | Now, imagine that you are invited to a group on Facebook by your colleagues.  When you join, you realize that the majority of people in this group are **different from you** in your likes and dislikes about the things that you just provided your answers to. In other words, they **do not share** your sense of humor, favorite types of movies to watch, how they spend their free time, as well as in ideology and political leanings. |
| Mixed | Now, imagine that you are invited to a group on Facebook by your colleagues.  When you join, you realize that **some people in this group are similar to you and some people are different from you** in your likes and dislikes about the things that you just provided your answers to. In other words, **some people share**your sense of humor, favorite types of movies to watch, how they spend their free time, as well as in ideology and political leanings, but **other people do not**. |
| Unclear | Now, imagine that you are invited to a group on Facebook by your colleagues.  When you join,**you aren't sure** whether people in this group are similar to you in your likes and dislikes about the things that you just provided your answers to. In other words, you **aren't sure**whether they share your sense of humor, favorite types of movies to watch, how they spend their free time, as well as in ideology and political leanings. |

References

1. Baek, E. C. *et al.* In-degree centrality in a social network is linked to coordinated neural activity. *Nat. Commun.* **13**, 1118 (2022).

2. Baek, E. C., Hyon, R., Lopez, K., Porter, M. A. & Parkinson, C. Lonely individuals process the world in idiosyncratic ways. *Psychol. Sci.* **34**, 683–695 (2023).

3. Chen, G. *et al.* Untangling the relatedness among correlations, part III: Inter-subject correlation analysis through Bayesian multilevel modeling for naturalistic scanning. *NeuroImage* **216**, 116474 (2020).
