# Supplementary material for: Perceived community alignment increases information sharing
Source: Nat Commun. 2025 Jul 1;16:5864. doi: 10.1038/s41467-025-59915-8 (PMC12215788; doi:10.1038/s41467-025-59915-8)
Supplement: Supplementary file 2 — Reporting Summary [file 41467_2025_59915_MOESM2_ESM.pdf]

nature portfolio

Corresponding author(s): Elisa C. Baek

Last updated by author(s): April 29, 2025

Reporting Summary

Nature Portfolio wishes to improve the reproducibility of the work that we publish. This form provides structure for consistency and transparency in reporting. For further information on Nature Portfolio policies, see our Editorial Policies and the Editorial Policy Checklist.

Statistics

For all statistical analyses, confirm that the following items are present in the figure legend, table legend, main text, or Methods section.

n/a	Confirmed	
☐	☒	The exact sample size (*n*) for each experimental group/condition, given as a discrete number and unit of measurement
☐	☒	A statement on whether measurements were taken from distinct samples or whether the same sample was measured repeatedly
☐	☒	The statistical test(s) used AND whether they are one- or two-sided<br>*Only common tests should be described solely by name; describe more complex techniques in the Methods section.*
☐	☒	A description of all covariates tested
☐	☒	A description of any assumptions or corrections, such as tests of normality and adjustment for multiple comparisons
☐	☒	A full description of the statistical parameters including central tendency (e.g. means) or other basic estimates (e.g. regression coefficient) AND variation (e.g. standard deviation) or associated estimates of uncertainty (e.g. confidence intervals)
☐	☒	For null hypothesis testing, the test statistic (e.g. *F*, *t*, *r*) with confidence intervals, effect sizes, degrees of freedom and *P* value noted<br>*Give P values as exact values whenever suitable.*
☒	☐	For Bayesian analysis, information on the choice of priors and Markov chain Monte Carlo settings
☐	☒	For hierarchical and complex designs, identification of the appropriate level for tests and full reporting of outcomes
☐	☒	Estimates of effect sizes (e.g. Cohen's *d*, Pearson's *r*), indicating how they were calculated

Our web collection on statistics for biologists contains articles on many of the points above.

Software and code

Policy information about availability of computer code

Data collection	Study 1: fMRI data were collected using a 3T Siemens Prisma scanner with a 32-channel coil.<br>Study 2: Behavioral data were collected using Qualtrics.<br>Study 3: Behavioral data were collected using Qualtrics.
Data analysis	Study 1: We used fMRIPrep version 1.4.0 for processing of our fMRI data. We calculated inter-subject correlations using SciPy in Python 3.7. Statistical analyses (GLMs and mixed-effects models) were conducted in R using emmeans and lmer packages.<br>Study 2: Statistical analyses (mixed-effects models) were conducted in R using the lmer package.<br>Study 3: Statistical analyses (GLMs) were conducted in R using emmeans and lm packages.<br><br>The code that we used in Study 1 is available at https://zenodo.org/records/15080347. The code that we used for Study 2 is available at https://zenodo.org/records/15020757. The code that we used for Study 3 is available at https://zenodo.org/records/15080343.

For manuscripts utilizing custom algorithms or software that are central to the research but not yet described in published literature, software must be made available to editors and reviewers. We strongly encourage code deposition in a community repository (e.g. GitHub). See the Nature Portfolio guidelines for submitting code & software for further information.

Data

Policy information about availability of data

All manuscripts must include a data availability statement. This statement should provide the following information, where applicable:

- Accession codes, unique identifiers, or web links for publicly available datasets
- A description of any restrictions on data availability
- For clinical datasets or third party data, please ensure that the statement adheres to our policy

The preprocessed data for Study 1 are available at https://doi.org/10.5281/zenodo.15080347, the preprocessed data and hyperlinks to the stimuli for Study 2 are available at https://doi.org/ https://doi.org/10.5281/zenodo.11432270, and the preprocessed data and hyperlinks to the stimuli for Study 3 are available at https://doi.org/10.5281/zenodo.15080343. Raw data can be obtained by contacting the corresponding authors.

Research involving human participants, their data, or biological material

Policy information about studies with human participants or human data. See also policy information about sex, gender (identity/presentation), and sexual orientation and race, ethnicity and racism.

Reporting on sex and gender

Study 1: We only collected participants' self-reported gender. There were 41 participants identifying as female and 25 participants identifying as male.
Study 2: We did not obtain any information about the participants' gender or sex.
Study 3: We only collected participants' self-reported gender. There were 223 participants identifying as female, 68 participants identifying as male, 8 participants who reported "other", and 1 participant who responded "would rather not say".

We did not consider sex or gender in the study design, and we did not conduct any sex or gender analysis because this is not a focal part of the study.

Reporting on race, ethnicity, or other socially relevant groupings

Study 1: Participants self-reported their race and were able to select multiple races out of the following: American Indian or Alaska Native, Asian, Black or African American, Hispanic or Latinx, Native Hawaiian or Other Pacific Islander, White (not Hispanic or Latinx), Other. The number of participants' self-reported race(s) is below. Please note that some participants self-reported as identifying with more than one race.

American Indian/Alaska Native: 0
Asian: 24
Black/African American: 2
Hispanic/Latinx: 25
Native Hawaiian/Other Pacific Islander: 0
White (not Hispanic or Latinx): 24
Other: 0

Study 2: We did not obtain any information about the participants' race or ethnicity.

Study 3: Participants self-reported their race and were able to select multiple races out of the following: American Indian or Alaska Native, Asian, Black or African American, Hispanic or Latinx, Native Hawaiian or Other Pacific Islander, White (not Hispanic or Latinx), Other. The number of participants' self-reported race(s) is below. Please note that some participants self-reported as identifying with more than one race.

American Indian/Alaska Native: 2
Asian: 24
Black/African American: 21
Hispanic/Latinx: 27
Native Hawaiian/Other Pacific Islander: 0
White (not Hispanic or Latinx): 240
Other: 3

Population characteristics

See below.

Recruitment

Study 1: The study research sample consists of first-year undergraduate students living in two different residential communities at a large state university (specifically, at University of California, Los Angeles) in the United States. Participants were recruited through flyers and advertisement through email listservs. Given that Study 1's scientific aim was to understand whether stimuli that are similarly interpreted across individuals in the same social community are also more likely to be shared, we encouraged interested participants to reach out to other residents in their residence hall and fill out a survey. Two residential communities with the highest level of participation were selected for participation in the study.

Study 2: Participants were recruited using Amazon's Mechanical Turk.

Study 3: Participants were recruited on Prolific.

Participants across all three studies were unaware of the aims of the study, and participants in Study 3 were randomly assigned to the experimental condition. Thus, it is unlikely that a self-selection bias occurred.

Ethics oversight	All studies were approved by the Institutional Review Board of the University of California, Los Angeles. Study 1 participants provided informed consent in accordance with the procedures of the Institutional Review Board of the University of California, Los Angeles. Study 2 and Study 3 were certified exempt.

Note that full information on the approval of the study protocol must also be provided in the manuscript.

Field-specific reporting

Please select the one below that is the best fit for your research. If you are not sure, read the appropriate sections before making your selection.

☐ Life sciences ☒ Behavioural & social sciences ☐ Ecological, evolutionary & environmental sciences

For a reference copy of the document with all sections, see nature.com/documents/nr-reporting-summary-flat.pdf

Behavioural & social sciences study design

All studies must disclose on these points even when the disclosure is negative.

Study description	All three studies are quantitative studies that investigate the associations between perceived similarity and sharing likelihood.
Research sample	Study 1: The study research sample consists of first-year undergraduate students living in two different residential communities at a large state university (specifically, at University of California, Los Angeles) in the United States. Given that our key research question asked whether participants were more likely to share content that would be interpreted similarly, as indicated by similar neural responses, by others in their social community, our sample of individuals living in the same social communities is appropriate for our core scientific research question of interest. One potential concern is that our findings would not generalize across different age groups or social contexts; however, our follow-up behavioral studies (Studies 2 and 3) that directly test the mechanisms that we identified using neuroimaging in Study 1 consist of participants who were recruited online and therefore represent more diverse populations.<br><br>Study 2: The study research sample consists of online participants who were recruited on Amazon's Mechanical Turk. Participants were required to be fluent in English and living in the United States, as well as pass a basic English comprehension check in order to participate. We did not obtain any demographic information from Study 2.<br><br>Study 3: The study research sample consists of online participants who were recruited on Prolific. Participants were required to be fluent in English and living in the United States, as well as be regular users of Facebook (defined as using it at least once a month). Given that Study 3 instructed participants to consider sharing information with potential Facebook groups, our method of ensuring that participants were regular Facebook users is appropriate. Participants ranged in age with a minimum age of 19, a maximum age of 82, a mean age of M = 36.69, and a standard deviation of SD = 13.87.<br><br>None of the study samples were nationally representative, and our Study 1 sample consists of young adults and Studies 2 and 3 samples consist of participants that were recruited online. Accordingly, it is possible that the study results would not generalize to individuals who do not use the Internet regularly; however, given that our scientific question of interest investigates online information sharing, our research samples are appropriate.
Sampling strategy	Study 1: We sought to recruit as many participants as possible who were living in the two different residential communities at the University of California, Los Angeles. This resulted in a total of 64 participants (after exclusions) who were included for analyses. Our final N size of 63 participants for all analyses is larger than previously published studies investigating the relationships between ISCs and behavioral outcomes (e.g., Parkinson, Kleinbaum & Wheatley, Nature Comms, 2018; Finn et al., Nature Comms, 2018).<br><br>Study 2: Participants were recruited on Amazon's Mechanical Turk. The sample size was determined by a power analysis based on pilot data, which suggested that a sample size of 100 participants would have 95% power to detect a standardized effect size of d = 0.13, which was the smallest estimated effect size based on pilot data.<br><br>Study 3: Participants were recruited on Prolific. The sample size was determined by a power analysis based on pilot data, which suggested that a sample size of 300 participants would have 85% power to detect a standardized effect size of d = 0.25, which was the smallest estimated effect size based on pilot data.
Data collection	Study 1: Neuroimaging data were collected using a 3T Siemens Prisma scanner with a 32-channel coil, and two researchers were present for all data analysis (due to the safety protocol of the fMRI scanning center). The researchers were not aware of the hypotheses during data collection. The sharing ratings were collected on a research laptop in an isolated room in the fMRI scanning center, and researchers were not inside the room while participants completed the surveys.<br><br>Study 2: Participants completed the experiment online via their own computers.<br><br>Study 3: Participants completed the experiment online via their own computers.
Timing	Study 1: Data collection occurred between September and early November of 2019.<br>Study 2: Data collection began and finished in January 2021.<br>Study 3: Data collection began and finished in June 2022.
Data exclusions	Study 1: A total of 70 participants from the two residential communities participated in the neuroimaging portion of our study. We excluded four subjects from the fMRI data that we analyzed; two subjects had excessive movement in more than half of the scan,

one subject fell asleep during half of the scan, and one subject did not complete the scan. Exclusion criteria were pre-established.

Study 2: All participants were required to meet our eligibility criteria in order to participate in the study. Eligibility criteria were pre-established and pre-registered. Accordingly, we did not exclude any participants who completed the study.

Study 3: All participants were required to meet our eligibility criteria in order to participate in the study. Eligibility criteria were pre-established and pre-registered. Accordingly, we did not exclude any participants who completed the study.

Non-participation

Study 1: One participant ended the fMRI scanning session early.
Study 2 and Study 3: No participants dropped out.

Randomization

Study 1 and Study 2: Participants were not allocated into experimental groups.
Study 3: Participants were uniformly randomly assigned to experimental groups.

Reporting for specific materials, systems and methods

We require information from authors about some types of materials, experimental systems and methods used in many studies. Here, indicate whether each material, system or method listed is relevant to your study. If you are not sure if a list item applies to your research, read the appropriate section before selecting a response.

Materials & experimental systems

n/a	Involved in the study
☒	☐ Antibodies
☒	☐ Eukaryotic cell lines
☒	☐ Palaeontology and archaeology
☒	☐ Animals and other organisms
☒	☐ Clinical data
☒	☐ Dual use research of concern
☒	☐ Plants

Methods

n/a	Involved in the study
☒	☐ ChIP-seq
☒	☐ Flow cytometry
☐	☒ MRI-based neuroimaging

Magnetic resonance imaging

Experimental design

Design type

Task fMRI during viewing of naturalistic stimuli

Design specifications

Participants watched 14 different videos in the fMRI scanner. The fMRI study was divided into four runs and lasted approximately 60 minutes in total.

Behavioral performance measures

Given that participants were instructed to passively view the stimuli, no behavioral performance measures were collected.

Acquisition

Imaging type(s)

functional, structural

Field strength

3T

Sequence & imaging parameters

The participants were scanned using a 3T Siemens Prisma scanner with a 32-channel coil. Functional images were recorded using an echo-planar sequence (with echo time = 37 ms, repetition time = 800 ms, voxel size = 2.0 mm × 2.0 mm × 2.0 mm, matrix size = 104 × 104 mm, field of view = 208 mm, slice thickness = 2.0 mm, multi-band acceleration factor = 8, and 72 interleaved slices with no gap). A black screen was included at the beginning (with duration = 8 seconds) and the end (duration = 20 seconds) of each run to allow the BOLD signal to stabilize. We also acquired high-resolution T1-weighted (T1w) images (with echo time = 2.48 ms, repetition time = 1,900 ms, voxel size = 1.0 mm × 1.0 mm × 1.00 mm, matrix size = 256 × 256 mm, field of view = 256 mm, slice thickness = 1.0 mm, and 208 interleaved slices with 0.5 mm gap) for coregistration and normalization. We attached adhesive tape to the head coil in the MRI scanner and applied it across the participants' foreheads, which has been shown to significantly reduce head motion.

Area of acquisition

A whole brain scan was used.

Diffusion MRI ☐ Used ☒ Not used

Preprocessing

Preprocessing software

We used fMRIPrep version 1.4.0 for the data processing of our fMRI data. We have taken the descriptions of anatomical and functional data preprocessing that begins in the next paragraph from the recommended boilerplate text that is generated by fMRIPrep and released under a CC0 license, with the intention that researchers reuse the text to facilitate clear and

	consistent descriptions of preprocessing steps, thereby enhancing the reproducibility of studies.<br>For each subject, the T1-weighted (T1w) image was corrected for intensity non-uniformity (INU) with N4BiasFieldCorrection, distributed with ANTs 2.1.0, and used as T1w-reference throughout the workflow. Brain tissue segmentation of cerebrospinal fluid (CSF), white matter (WM) and gray matter (GM) was performed on the brain-extracted T1w using FSL fast. Volume-based spatial normalization to the ICBM 152 Nonlinear Asymmetrical template version 2009c (MNI152NLin2009cAsym) was performed through nonlinear registration with antsRegistration (ANTs 2.1.0).<br>For each of the four BOLD runs per participant, the following preprocessing was performed. First, a reference volume and its skull-stripped version were generated using a custom methodology of fMRIPrep. The BOLD reference was then coregistered to the T1w reference using FSL flirt with the boundary-based registration cost function. The coregistration was configured with nine degrees of freedom to account for distortions remaining in the BOLD reference. Head-motion parameters with respect to the BOLD reference (transformation matrices, and six corresponding rotation and translation parameters) were estimated before any spatiotemporal filtering using FSL mcflirt. Automatic removal of motion artifacts using independent component analysis (ICA–AROMA) was performed on the preprocessed BOLD on MNI space time series after removal of non-steady state volumes and spatial smoothing with an isotropic, Gaussian kernel of 6mm FWHM (full-width half-maximum). The BOLD time series were then resampled to the MNI152Nlin2009cAsym standard space.<br>The following 10 confounding variables generated by fMRIPrep were included as nuisance regressors: global signals extracted from within the cerebrospinal fluid, white matter, and whole-brain masks, framewise displacement, three translational motion parameters, and three rotational motion parameters.
Normalization	Volume-based spatial normalization to the ICBM 152 Nonlinear Asymmetrical template version 2009c (MNI152NLin2009cAsym) was performed through nonlinear registration with antsRegistration (ANTs 2.1.0).
Normalization template	ICBM 152 Nonlinear Asymmetrical template version 2009c (MNI152NLin2009cAsym)
Noise and artifact removal	Head-motion parameters with respect to the BOLD reference (transformation matrices, and six corresponding rotation and translation parameters) were estimated before any spatiotemporal filtering using FSL mcflirt. Automatic removal of motion artifacts using independent component analysis (ICA–AROMA) was performed on the preprocessed BOLD on MNI space time series after removal of non-steady state volumes and spatial smoothing with an isotropic, Gaussian kernel of 6mm FWHM (full-width half-maximum). The following 10 confounding variables generated by fMRIPrep were included as nuisance regressors: global signals extracted from within the cerebrospinal fluid, white matter, and whole-brain masks, framewise displacement, three translational motion parameters, and three rotational motion parameters.
Volume censoring	Not applicable.

Statistical modeling & inference

Model type and settings	We calculated inter-subject correlations (ISCs) of time series of neural responses that were measured with fMRI to capture shared neural responses across subjects during the processing of naturalistic stimuli (see Fig. 1). First, we extracted the mean-response time series across the entire video-viewing task from both (1) each of the 200 cortical parcels in the 200-parcel version of the Schaefer et al. (2018) parcellation scheme and (2) 14 subcortical parcels in the Harvard-Oxford subcortical atlas. This resulted in a total of 214 brain regions across the whole brain. For each of the 2,145 unique pairs of participants (i.e., dyads) in our fMRI sample, we then computed the Pearson correlation between the dyad members' time series of neural responses for each cortical parcel. This yields one correlation coefficient per unique dyad for each brain parcel. We then used fit linear mixed-effects models with crossed random effects to test our hypotheses.
Effect(s) tested	We tested the relationships between ISCs and participants' self-reported likelihood of sharing.

Specify type of analysis: ☒ Whole brain ☐ ROI-based ☐ Both

Statistic type for inference<br>(See Eklund et al. 2016)	As described in the "Model type and settings" field above, our analyses compare responses within each of 214 anatomically-defined brain regions, and thus are not impacted by the concerns that the Eklund et al. (2016) paper raised regarding inflated false-positive rates in fMRI inferences for spatial extent.<br><br>Correction for multiple comparisons across brain regions was implemented using False-Discovery Rate (FDR) correction (as specified in the "Correction" field below).
Correction	We used Holm-Bonferroni correction for all analyses.

Models & analysis

n/a	Involved in the study
☒	☐ Functional and/or effective connectivity
☒	☐ Graph analysis
☒	☐ Multivariate modeling or predictive analysis
